# Attracting private sector inflows to close the financing gap for universal health coverage: What questions need to be answered?

**DOI:** 10.7189/jogh.13.03013

**Published:** 2023-04-28

**Authors:** Brendan Kwesiga, Regina Titi-Ofei, Juliet Nabyonga-Orem

**Affiliations:** 1 World Health Organization, Universal Health Coverage and Life Course Cluster, Nairobi, Kenya; 2 World Health Organization, Office of the Assistant Regional Director, Brazzaville, Congo; 3 World Health Organization, Universal Health Coverage and Life Course Cluster, Brazzaville, Congo; 4 Centre for Health Professions Education, Faculty of Health Sciences, North-West University, Potchefstroom, South Africa

A financial gap exists between the resources available for Universal Health Coverage (UHC) and current needs [1]. Even the available resources have often been misused [2], exacerbating existing inequities by widening the access gap. To address this challenge, calls have been issued to leverage the private sector to close both the financing and access gaps. The need for health systems to attract additional private sector resources has been highlighted, as donor funding continues to decrease, with the trend expected to worsen because of the impact of COVID-19 on countries providing donor aid. The Addis Ababa Action Agenda of the 3^rd^ International Conference on Financing for Development emphasized the need to identify mechanisms for unlocking the potential of private sector investment by incentivizing changes in financing to support sustainable development [3]. However, while there are legitimate concerns about how to urgently raise additional funds to close the UHC financing and access gaps, there is a need to ensure that the mechanisms and instruments used to achieve this are informed by relevant country- and context-specific evidence and aligned to the overall aspirations of UHC by 2030 [4].

Proponents of the increased role of private sector investment in UHC contend that it delivers a shared value of a “triple win” for countries. The argument is that governments are able to obtain maximum benefit from limited public capital, the population is able to obtain higher-quality health services at the same or lower cost, and private players obtain a sustainable return on their investment and expertise [5]. Consequently, they conclude that private investment is a panacea that should complement or even replace “traditional” financing models. However, opponents argue that private sector investment in health is a rent-seeking avenue for the privatization of profits and the socialization of losses. They argue that economic exigencies and private interests increasingly erode gains of publicly financed UHC, built on sustained public sector underinvestment that perpetuates further dysfunction, leading to increased incentivization of private sector expansion [6,7]. This exacerbates inequities within the health system, as private sector interests may provide strong resistance to policies meant to address inequities.

**Figure:**
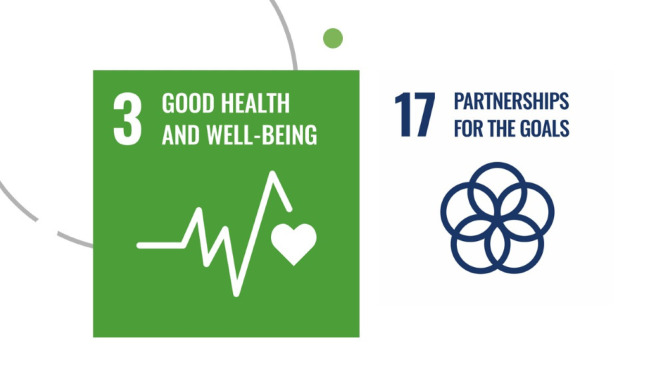
Photo: Forging the right partnership in financing universal health coverage. Modified by authors from free-to-use photos offered at the United Nations’ website, available at: https://www.un.org/sustainabledevelopment/news/communications-material/.

These contrasts show that, for interventions for crowding in private finance in health to be scalable, they need to be based on clear evidence and must catalyse progress to UHC goals, with a clear understanding of the risks and consequences in different contexts. Within SDG 3, the UHC goal is considered paramount and should provide the normative framework on which mechanisms for attracting private sector investments in the health sector can be appraised. The UHC goal is associated with international mandates through numerous United Nations General Assembly and World Health Assembly resolutions [8,9]. WHO has also developed technical guidance on how countries can orient their health systems financing towards the UHC goals [10,11].

## OVERVIEW OF THE FRAMEWORK FOR FINANCING HEALTH SYSTEMS FOR UNIVERSAL HEALTH COVERAGE

The 2010 World Health Report (WHR), “Health systems financing: The Path to Universal Coverage”, introduced a policy construct of “Health Financing for Universal Health Coverage”. The report declared the aspiration for financing systems to be able to “provide all people with access to needed health services of sufficient quality to be effective; ensure that the use of these services does not expose the user to financial hardship” [2]. This report explicitly outlines the instruments, institutions, and incentives required to organize the collection of financial resources, and their pooling and flow from source to provider within the health system, to move towards UHC [2]. It has also formed the basis of other subsequent political declarations and technical framing around financing for UHC.

Despite these political drivers that have positioned health system financing as critical to the attainment of UHC, most of the literature on financing for UHC by WHO and health financing policy practitioners has mainly focused on recurrent expenses/costs and not capital costs. While there is a need for more dedicated focus on how to finance capital investment in health, this paper also focuses on recurrent costs.

## OVERVIEW OF BLENDED FINANCING IN THE HEALTH SECTOR

Blended financing, where development and philanthropic funds are used to catalyze additional capital inflows to the health sector, has been heralded as one of the potential innovative solutions for unlocking private sector investment in health [12-14]. · This approach aims at attracting commercial capital towards projects/programs that benefit society, while also providing financial returns to investors [15]. It focuses on combining financing sources that have a developmental mandate with those that have a commercial mandate, while also reshaping the traditional partnership models between the public and private sectors by focusing on outcome-oriented incentives [12]. It is a structuring approach where public and private entities with different objectives invest alongside each other to achieve their own objectives (whether financial return, social impact, or a blend of both). Blended financing instruments differ in management and transaction types/vehicles, depending on the program being financed and the intended incentive [16]. The different blended financing tools used in the health sector are summarised in **Table 1**.

**Table 1:** Blended financing instruments for the health sector, adapted from USAID [13]

Instrument	Description	What is incentivized?	Who is incentivized?	Who pays?
Credit guarantees and de-risking	Public funding or combination of debt, equity, and guarantees from ODA or MDBs used to provide guarantees or insurance, which can reduce the investment risk faced by private investors and/or improve the expected returns from an investment in a health focused project.	Increase private finance, given use of concessionary capital to absorb risk of private sector investments. Mechanisms include “first loss” grants, guarantees, and subordinated debt.	Private sector, given absorption of risk by public sector and development partners.	Private investors
Development impact bonds	Result based financing instruments that bring together public and private funding, and link payouts to the attainment of pre-determined, measurable health outcomes.	Delivery of results by the service providers. health Sector outcome payers are usually development agencies/donors, investors are private sector and service provider is identified provider from health sector.	Investor for the risk of investment and opportunity for a risk-adjusted return paid by outcome payer.	Private investors
Loan buy downs	Donor funds used to pay part or all the principal or interest on a loan on behalf of a recipient country, contingent on achievement of predetermined milestones or outcomes.	Increased government/domestic investment in health.	ODA/multilateral concessional loan recipient governments. Incentive to increase investment in domestic public health programs.	Donor agency/partner

Official development assistance – ODA, Multilateral Development Bank – MDB

The main blended finance instruments that have been used to attract private inflows for the health sector are impact bonds [17]. Impact bonds are innovative performance-based contracts between an investor, an outcome funder, and a service provider that tackle a social challenge [17]. With the growing implementation of blended financing in the health sector in developing countries, there is a need to understand how blended finance instruments can best be used in low- and middle-income countries (LMICs) [18]. Although the implementation of blended finance is growing, the field of evaluation is still nascent, especially in low-income countries [19,20]. We will now propose some areas that are key for not only developing impact bonds, but also for other innovative financing tools aimed at attracting private sector inflows into the health sector.

## WHAT QUESTIONS NEED TO BE ADDRESSED?

For blended finance instruments to improve ways of channelling increased and better-quality financial flows in low-income health systems, their design and implementation need to be informed by clear evidence. The conventional development finance metrics used to evaluate blended financing are leverage (additional commercial/non-concessional capital inflows attracted), impact (additional impact achieved due to the private sector inflow) and financial return on investment (for the private sector). We argue that these measures of success are not health systems-oriented and may not adequately address health policymakers’ concerns on impact and improved health outcomes. While it is usually clear for private sector investors why they are engaging in a “deal” that requires them to invest in a health intervention, the decision about this is not always that straightforward for policymakers. Based on this, we propose some questions to guide the reflections of a health policy maker, in considering private sector inflows to the health sector.

### What works and in which context?

This has been shown to be a major gap in the blended financing literature [21] and has hardly been addressed in the case of the application of blended financing to the health sector in LMICs. Using a realist evaluation approach, one can be able to explore “what makes a blended financing intervention work (or not)?”, the context in which these may be triggered (what are the enabling factors/conditions in which an intervention is effective?). and how these trigger mechanisms influence the attainment of the desired outcomes [22]. This needs to consider both the technical and non-technical conditionalities (institutional, legal, and political conditions) that need to be in place for successful implementation.

### Does it render a comparative advantage/concrete value addition over traditional financing approaches?

Given that, in several contexts, blended financing relies on the use of public/concessional financing to attract private sector inflows, Barder and Talbot [16] have argued that blending should only be used when it is recognized as the most preferable way of funding a specific problem. While it is (or should be) obvious that public funds are used for health interventions of known cost-effectiveness, the use of blending also requires that the combination of risk and returns would have been unable to attract private sector investment (hence the need for blending). If blending is done for an intervention with competitive risk returns, it results in extra profit for private investors and crowds out potentially additional private investment. This is because public funds would have been used for investments that the private sector would have made in any case, given the competitive return. Therefore, while private sector investors may be interested in the return on investment (i.e. the expectation that the transaction should achieve a positive financial return), there is a need for the public sector to consider whether the current funding landscape requires blending to catalyse improved health outcomes.

### Under what circumstances are we able to achieve financial additionality?

This question has been widely discussed in the blended finance evaluation literature [19]. However, given that the overall aim of blending is not just achieving additional private sector inflows but also ensuring that this translates into outcome and impact additionality, there is a need to compare the “incremental cost per outcome” for blended financing with the “status quo” (public financing only approach). This would help answer the question of whether we should be using blended financing for that specific intervention. This question should be based on both the short/immediate-term and the long-term, in case intervention costs can be reduced over time (based on increased coverage/demand and economies of scale) so that there is a reduced need to rely on concessional funds or when outcomes are expected long after the investment. These questions are also important for addressing sustainability and scalability issues, especially when blending, and may rely on donor funding.

## CONCLUSION

There is no doubt that achieving more sustainable financing in light of the 2030 SDG agenda will require significant additional investments. However, as countries attempt to achieve the ambitious UHC goals, particularly considering resource constraints and shrinking fiscal space, there is a temptation to jump at anything labelled “innovative”, while in other instances, extreme cynicism may make practitioners “throw the baby out with the bathwater”. This paper highlights some key questions for both design and implementation that policymakers in resource-constrained countries may have to consider as they reflect on the adoption of instruments for attracting additional private sector investment.
